# Multiple measures could alleviate long-branch attraction in phylogenomic reconstruction of Cupressoideae (Cupressaceae)

**DOI:** 10.1038/srep41005

**Published:** 2017-01-25

**Authors:** Xiao-Jian Qu, Jian-Jun Jin, Shu-Miaw Chaw, De-Zhu Li, Ting-Shuang Yi

**Affiliations:** 1 Germplasm Bank of Wild Species, Kunming Institute of Botany, Chinese Academy of Sciences, Kunming, Yunnan 650201, China; 2 Kunming College of Life Sciences, University of Chinese Academy of Sciences, Kunming, Yunnan 650201, China; 3 Biodiversity Research Center, Academia Sinica, Nankang District, Taipei 11529, Taiwan

## Abstract

Long-branch attraction (LBA) is a major obstacle in phylogenetic reconstruction. The phylogenetic relationships among *Juniperus* (J), *Cupressus* (C) and the *Hesperocyparis-Callitropsis-Xanthocyparis* (HCX) subclades of Cupressoideae are controversial. Our initial analyses of plastid protein-coding gene matrix revealed both J and C with much longer stem branches than those of HCX, so their sister relationships may be attributed to LBA. We used multiple measures including data filtering and modifying, evolutionary model selection and coalescent phylogenetic reconstruction to alleviate the LBA artifact. Data filtering by strictly removing unreliable aligned regions and removing substitution saturation genes and rapidly evolving sites could significantly reduce branch lengths of subclades J and C and recovered a relationship of J (C, HCX). In addition, using coalescent phylogenetic reconstruction could elucidate the LBA artifact and recovered J (C, HCX). However, some valid methods for other taxa were inefficient in alleviating the LBA artifact in J-C-HCX. Different strategies should be carefully considered and justified to reduce LBA in phylogenetic reconstruction of different groups. Three subclades of J-C-HCX were estimated to have experienced ancient rapid divergence within a short period, which could be another major obstacle in resolving relationships. Furthermore, our plastid phylogenomic analyses fully resolved the intergeneric relationships of Cupressoideae.

Rapid radiation of a clade, especially with longer external branches relative to internal branches, is a major obstacle in phylogenetic reconstruction^1^,^2^,^3^. Homoplastic mutations occurring along the long external branches can obscure the true phylogenetic signal on the internal branches, then long-branch attraction (LBA) occurs, with the long branches erroneously clustered together^4^,^5^. The LBA artifact was suggested to adversely affect the accuracy of tree reconstruction in many phylogenetic studies^6^,^7^,^8^,^9^,^10^,^11^. It could be amplified in phylogenomics, thereby resulting in a strongly supported but incorrect phylogeny^6^,^12^.

Major factors contributing to LBA include faster substitution rate in nonadjacent phylogenetic lineages^4^, poor taxon sampling due to extinction or unavailability of some taxa^13^, and unsuitable models of sequence evolution accounting for base compositional heterogeneity^14^,^15^ and lineage-specific changes^16^. Multiple strategies proposed to alleviate the LBA artifact include increasing the representation of taxon sampling^5^,^10^,^17^,^18^,^19^; removing the long branch^5^,^9^,^20^, although this method cannot be applied to a trichotomy; excluding third codon positions^21^; using amino acids instead of nucleotides^22^,^23^,^24^; removing unreliable aligned regions^25^; removing rapidly evolving genes or sites^7^,^10^,^26^,^27^,^28^; applying the site-heterogeneous CAT model;^24^,^29^,^30^,^31^ and applying coalescent-based tree-building methods^32^,^33^.

The cypress family, Cupressaceae, contains about 140 species in 32 genera (17 monotypic) with worldwide distribution^34^,^35^,^36^,^37^. Recent molecular phylogenetic studies have divided this family into seven subfamilies^35^,^36^,^38^. Five subfamilies (i.e., Cunninghamioideae, Cupressoideae, Sequoioideae, Taiwanioideae and Taxodioideae) are distributed solely in the Northern Hemisphere, and Athrotaxidoideae and Callitroideae are indigenous to the Southern Hemisphere. Cupressoideae, the largest subfamily of Cupressaceae, contains about 100 species in 13 genera^34^,^35^,^36^,^37^. Species of Cupressoideae are important in the North Hemisphere forest, and many are constructive and dominant species. Many species are also economically important as timber sources, especially species of *Calocedrus, Chamaecyparis, Cupressus* and *Thuja*.

Because the Cupressoideae are an ecologically and economically important group, many phylogenetic studies have been performed to resolve intergeneric relationships^35^,^36^,^38^,^39^,^40^,^41^. With the application of several genetic markers and sufficient taxon sampling, two recent phylogenetic studies of Cupressaceae^35^,^36^ strongly supported Cupressoideae as monophyletic. Although these two studies resolved most of the intergeneric relationships, discrepancies among different gene trees were detected depending on the phylogenetic positions of the *Thuja-Thujopsis* and *Chamaecyparis-Fokienia* clades as well as relationships in the *Juniperus* (J)-*Cupressus* (C)-*Hesperocyparis-Callitropsis-Xanthocyparis* (HCX) clade (the J-C-HCX clade thereafter). In fact, the phylogenetic relationships among the three subclades of J-C-HCX have long been controversial in a series of phylogenetic studies; various relationships, including HCX (J, C), J (C, HCX) or C (J, HCX) were supported by different datasets and/or analyses^35^,^36^,^39^,^40^,^41^,^42^,^43^,^44^.

Plastid phylogenomics has been successfully used to determine difficult-to-resolve low-level relationships of plant groups^45^,^46^,^47^,^48^,^49^,^50^. In this study, we investigated 10 newly sequenced plastomes and seven previously published ones, representing 11 of the 13 genera in this subfamily, to resolve the intergeneric relationships of Cupressoideae. One or two representative species from each of the subfamilies Cunninghamioideae, Sequoioideae, Taiwanioideae and Taxodioideae were included as outgroups. Phylogenetic analyses based on original protein-coding genes (PCGs) suggested a sister relationship between *Juniperus* and *Cupressus* (Fig. 1). However, both *Juniperus* and *Cupressus* have long stem branches, so LBA may explain this relationship. In this case, plastid phylogenomics could be used to verify the effectiveness of the multiple strategies previously mentioned in alleviating the LBA artifact and reconstructing a robust Cupressoideae phylogeny. Comparative analyses based on data filtering and modifying, evolutionary model selection and coalescent phylogenetic reconstruction were used to explore efficient methods to alleviate the LBA artifact on the phylogenetic reconstruction of the J-C-HCX clade and resolve the intergeneric relationships of Cupressoideae.

## Results

### Sequence characteristics of data matrices

The PCG matrix has an aligned length of 81,423 bp with 11,394 bp parsimony-informative sites (Table 1). The characteristics of modified matrices from PCG by various filtering methods are in Table 1.

### Intergeneric relationships of Cupressoideae

**Figure 1:**
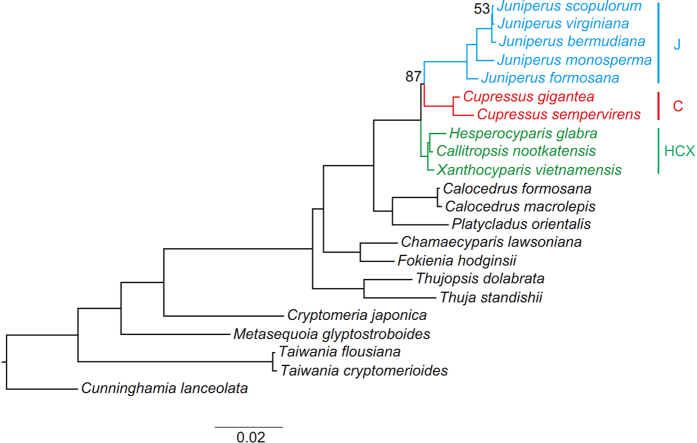
Phylogeny of Cupressoideae by maximum likelihood (ML) analysis of the PCG matrix under the GTRGAMMA model. Support for the branches was estimated from 1,000 bootstrapping replicates. The numbers on branches are bootstrap support values, and bootstrap values of 100% are not shown.

**Figure 2:**
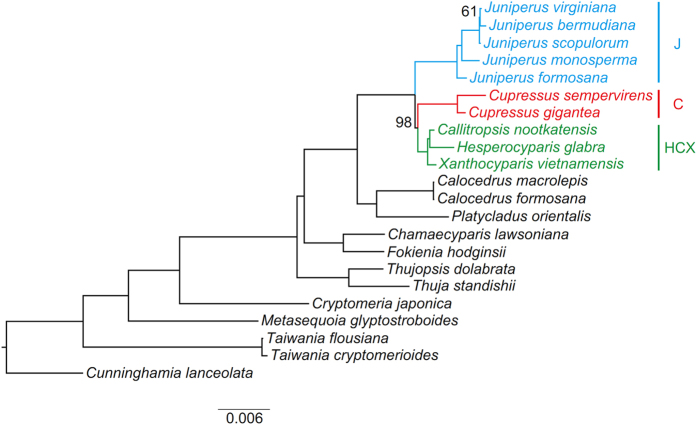
Phylogeny of Cupressoideae by the ML analysis of the PCG-Iss-73 matrix under the GTRGAMMA model. Support for the branches was estimated from 1,000 bootstrapping replicates. The numbers on branches are bootstrap support values, and bootstrap values of 100% are not shown.

The ML analysis of the PCG matrix fully resolved the intergeneric relationships of Cupressoideae (Fig. 1). *Thuja-Thujopsis* was resolved as the first diverged clade, and *Chamaecyparis-Fokienia* as second, then sister pairs of *Calocedrus-Platycladus* and J-C-HCX. Analyses of matrices including PCG-OV/TIGER-slow/medium-partition and PCG-OV-sorted showed low resolution on intergeneric relationships of Cupressoideae (Supplementary Fig. S1g,h). Other analyses of PCG12, AA, PCG-GB-relaxed/default/strict, PCG-Iss-9/73, PCG-slope-12/70, PCG-R^2^-11/71, PCG-OV/TIGER-fast-partition, PCG-GTR-CAT and PCG-ASTRAL recovered the same relationships as for PCG except relationships in J-C-HCX (Fig. 2; Supplementary Fig. S1).

**Figure 3:**
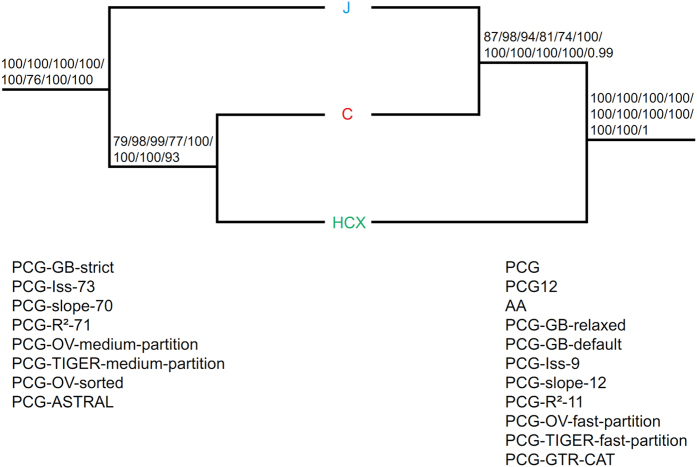
Reduced schematic trees showing different relationships and support inferred from all analyses. Two topologies resolved by analyses are shown. The left J (C, HCX) was supported by analyses of PCG-GB-strict, PCG-Iss-73, PCG-slope-70, PCG-R^2^-71, PCG-OV-medium-partition, PCG-TIGER-medium-partition, PCG-OV-sorted and PCG-ASTRAL, and bootstrap values from each analysis are shown on branches. The right HCX (J, C) was supported by analyses of PCG, PCG12, AA, PCG-GB-relaxed, PCG-GB-default, PCG-Iss-9, PCG-slope-12, PCG-R^2^-11, PCG-OV-fast-partition, PCG-TIGER-fast-partition and PCG-GTR-CAT, and bootstrap values are shown on branches. The complete topologies inferred from these datasets are in Figs 1 and 2 and Supplementary Fig. S1.

For J-C-HCX, HCX (J, C) was resolved in analyses of PCG, PCG12, AA, PCG-GB-relaxed/default, PCG-Iss-9, PCG-slope-12, PCG-R^2^-11, PCG-OV/TIGER-fast-partition, PCG-GTR-CAT (Figs 1 and 3; Supplementary Fig. S1a–h,j). However, a relationship of J (C, HCX) was supported in analyses of PCG-GB-strict, PCG-Iss-73, PCG-slope-70, PCG-R^2^-71, PCG-OV/TIGER-medium-partition, PCG-OV-sorted and PCG-ASTRAL (Figs 2 and 3; Supplementary Fig. S1c,e–h,k).

### Comparisons of branch lengths of subclades J, C and HCX in multiple analyses

**Figure 4:**
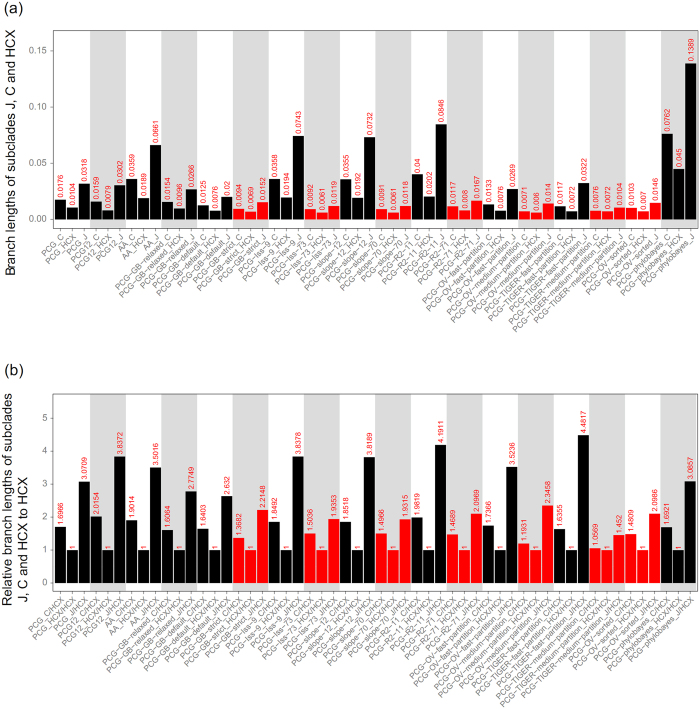
Comparison of branch lengths of subclades J, C and HCX in multiple analyses. The branch lengths for each of three subclades were calculated from the ML trees shown in Figs 1 and 2 and Supplementary Fig. S1. (**a**) Branch lengths for each subclade. The numbers above bars are branch length values. (**b**) Relative branch lengths of subclades J, C and HCX to HCX. The numbers above bars are the branch length ratio for each of three subclades relative to HCX.

Branch lengths of all three subclades were significantly reduced in analyses of PCG-GB-strict, PCG-Iss-73, PCG-slope-70 and PCG-R^2^-71 (Fig. 4a), and branch lengths of subclades J and C were reduced in analyses of PCG-OV/TIGER-medium-partition and PCG-OV-sorted (Fig. 4a). For analyses resolving J (C, HCX), the branch length ratio of subclades C to HCX ranged from 1.0569 to 1.5036 and ratio of J to HCX from 1.4520 to 2.3458 (Fig. 4b). For analyses resolving HCX (J, C), the branch length ratio of C to HCX ranged from 1.6064 to 2.0154 and ratio of J to HCX from 2.632 to 4.4817 (Fig. 4b).

### Divergence time estimations

**Figure 5:**
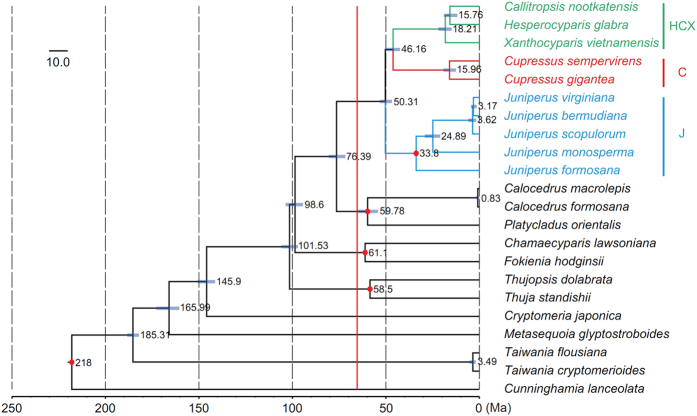
Chronogram of Cupressoideae based on the PCG-Iss-73 matrix inferred by using treePL. Blue bars represent the minimum and maximum estimation of node ages. Red dots represent fossil calibration points in Supplementary Table S3. Red line indicates the Cretaceous/Tertiary boundary.

The estimated divergence times based on matrices for PCG-Iss-73 (Fig. 5; Table 2) and PCG (Supplementary Fig. S5; Supplementary Table S4) were similar. By the PCG-Iss-73 matrix, the divergence time between subclades J and C-HCX was estimated at 50.31 million years ago (Ma; 46.84 to 53.34 Ma), and the split between C and HCX at 46.16 Ma (42.45 to 50.24 Ma) (Fig. 5; Table 2). By the PCG matrix, the divergence of subclades J-C from HCX was estimated at 48.79 Ma (47.48 to 50.29 Ma) and between subclades J and C at 47.64 Ma (46.16 to 48.87 Ma) (Supplementary Fig. S5; Supplementary Table S4). Our divergence time estimations are robust to topology differences.

## Discussion

Plastid phylogenomics fully resolved relationships among sampled genera of Cupressoideae with high support (Figs 1 and 2; Supplementary Fig. S1). We resolved *Thuja-Thujopsis* as diverging first, then *Chamaecyparis-Fokienia*. Our results agree with those of Mao *et al*.^35^ and most analyses of Yang *et al*.^36^. However, analyses of the nuclear LFY of Yang *et al*.^36^ resolved reverse relationships, and their mitochondrial rps3 resolved a monophyletic *Chamaecyparis-Fokienia* clade nested with a paraphyletic *Thuja-Thujopsis* clade. Our analyses of PCG-GB-strict, PCG-Iss-73, PCG-slope-70, PCG-R^2^-71, PCG-OV/TIGER-medium-partition, PCG-OV-sorted and PCG-ASTRAL resolved a relationship of J (C, HCX) (Figs 2 and 3; Supplementary Fig. S1c,e–i,k). This result agrees with previous analyses of plastid markers^35^,^36^,^42^ or combined plastid and nuclear matrix molecular analyses^35^,^41^. However, a relationship of HCX (J, C) was supported by the nuclear markers of Needly^36^,^40^, ABI3^42^ and Leafy^36^, the mitochondrial marker of rps3^36^, combined plastid markers^44^, and combined plastid and nuclear markers^36^. This topology was also recovered by some of our analyses involving original or slightly modified matrices or more complex models (Figs 1 and 3; Supplementary Fig. S1a–h,j). A relationship C (J, HCX) was also resolved by nrITS^39^,^40^,^42^,^44^, Needly^44^ and combined plastid and nuclear markers^42^,^44^.

Most previous analyses of plastid data except that of Terry and Adams^44^ revealed a relationship J (C, HCX). We also revealed this relationship with analyses of data matrices by removing unreliable aligned regions, substitution saturation genes and rapidly evolving sites or coalescent phylogenetic reconstruction on 82 aligned PCGs. The branch lengths of J and C were significantly reduced in these analyses (Fig. 4a). The branch length ratios of J and C relative to HCX in analyses of resolved J (C, HCX) were all much smaller than those in analyses of resolved HCX (J, C) (Fig. 4b). The LBA between subclades J and C and ancient rapid divergence [within 4.15 My between 46.16 and 50.31 Ma for J (C, HCX), or within 1.15 My between 48.79 and 47.64 Ma for HCX (J, C)] among three subclades (Fig. 5; Table 2; Supplementary Fig. S5; Supplementary Table S4) should jointly explain the long controversy about their relationships.

Filtering out unreliable alignment regions has been found critical for accurate phylogenetic inference^51^. Data filtering is sometimes efficient in reducing LBA in systematic analyses^25^. We recovered the relationship J (C, HCX) when using the strict strategy (PCG-GB-strict) (Supplementary Fig. S1c). Data filtering by using default or relaxed strategies (PCG-GB-relaxed/default) could also reduce the branch lengths of the subclades J and C to a certain degree (Fig. 4; Supplementary Fig. S1c); however, the resolved HCX (J, C) was probably due to unremoved homoplastic mutations along the stem of subclades J and C. In this study, data filtering by Gblocks was efficient in reducing the LBA artifact.

Rapidly evolving sites could accumulate multiple mutations, which tend to be saturated and contribute to LBA^52^,^53^,^54^. Although the plastome has usually been considered a single linked locus, processes such as recombination, gene conversion, heteroplasmy, and incomplete lineage sorting may also cause a heterogeneous evolutionary rate among different plastid genes^55^,^56^,^57^,^58^,^59^,^60^. The concatenated plastome dataset composed of subsets with different evolutionary rates could generate well-supported but conflicting phylogenetic topologies^32^,^61^. An effective way to reduce the effect of LBA is to remove rapidly evolving data^62^. For example, the phylogenetic position of Gnetales has long been controversial; the sister relationships between Gnetales and cupressophytes (the “Gnecup” hypothesis) seems to be caused by LBA. Removing rapidly evolving genes and three genes (psbC, rpl2 and rps7) that experienced many parallel amino acid substitutions supported a sister relationship between Gnetales and Pinaceae (the “Gnepine” hypothesis)^10^. The “Gnepine” hypothesis was also supported when excluding 2,250 of the mostly varied sites from the plastome matrix^27^. Removing saturated genes (PCG-Iss-73, PCG-slope-70, PCG-R^2^-71) significantly reduced branch lengths of the subclades J and C and recovered J (C, HCX) (Figs 2 and 4; Supplementary Fig. S1e,f). In addition, J (C, HCX) was supported when using the PCG-OV/TIGER-medium-partition including moderately evolving sites (Supplementary Fig. S1g,h). Furthermore, analysis of PCG-OV-sorted strongly favored J (C, HCX) when removing 4,250 mostly varied sites (Supplementary Fig. S1i). However, analyses of PCG-Iss-9, PCG-slope-12, PCG-R^2^-11 and PCG-OV/TIGER-fast-partition strongly supported HCX (J, C), which should be affected by LBA. In agreement with previous studies^10^,^27^,^28^,^54^,^63^, our results once again suggested LBA to be related to rapidly evolving sites.

Furthermore, we also found that the coalescent tree-building method (PCG-ASTRAL) could alleviate the LBA artifact in J-C-HCX. The coalescent method has been considered to better accommodate topological heterogeneity among gene trees^32^,^61^. This method is based on summary statistics calculated across all gene trees; a small number of outlier genes that significantly deviate from the coalescent model have relatively little effect on the accurate inference of the species tree^64^. The coalescent method may be efficient in reducing LBA^32^,^33^. Our coalescent analyses recovered J (C, HCX), which further supported the efficiency of this method in reducing the LBA artifact (Supplementary Fig. S1k).

Analyses of PCG12, AA and PCG-GTR-CAT have been found efficient in reducing LBA. More silent substitutions were suggested to occur at third codon positions, which should be downweighted in phylogenetic reconstruction^65^,^66^. Excluding third-codon positions has been used to reduce LBA and recovered monophyletic gymnosperms with Gnetales as sister to conifers^21^. In contrast, when including third-codon positions, gymnosperms were paraphyletic and Gnetales were inferred as sister to all other seed plants. Analyses of amino acids instead of nucleotides have been suggested to reduce systematic error introduced by substitutional saturation and to reduce LBA for reconstructing accurate phylogenetic relationships^22^,^23^,^24^. The site-heterogeneous mixture CAT model may be used to deal with heterotachy^67^ and has been found quite effective in overcoming the effect of LBA^24^,^29^,^30^,^31^. However, these three methods support a relationship of HCX (J, C) (Supplementary Fig. S1a,b,j), so they may not alleviate the effect of the LBA artifact in J-C-HCX.

Some previously reported methods have been efficient in reducing relative branch lengths of subclades J and C, and the LBA artifact was alleviated in phylogenetic reconstruction of J-C-HCX. However, some valid methods for other taxa are inefficient in reducing the LBA artifact and recovering HCX (J, C). As mentioned previously, LBA could be produced by complex sources. Different methods are appropriate for different groups. Multiple strategies should be carefully considered and justified to reduce LBA in phylogenetic reconstruction.

## Materials and Methods

### Taxon sampling

Young, fresh leaves were sampled from 10 species representing nine genera of the subfamily Cupressoideae from the Botanical Garden of the Kunming Institute of Botany (KIB), Chinese Academy of Sciences (CAS), or from the field in the island of Taiwan (Supplementary Table S1). Vouchers were deposited in the herbarium at KIB, CAS (KUN). A total of 22 Cupressaceae species representing five of seven subfamilies were included in this study, among which 17 species represent 11 of 13 genera of the subfamily Cupressoideae, with five species from the subfamilies Cunninghamioideae, Sequoioideae, Taiwanioideae and Taxodioideae used as outgroups (Supplementary Table S1). Because of inaccessibility, we were unable to obtain any samples of *Microbiota* or *Tetraclinis*. For J-C-HCX, we collected representative species for each of three genera of HCX; we also sampled two or more species to represent lineage diversification of *Cupressus* and *Juniperus* based on previous studies^35^,^41^.

### Plastid DNA extraction and sequencing

Pure plastid DNA was extracted from about 50 g of fresh leaves^68^,^69^. The concentration and sample integrity of the extracted plastid DNA were confirmed by using a Qubit Fluorometer (Beijing Genomics Institute [BGI], Shenzhen, Guangdong, China) and agarose gel electrophoresis. A 50 mg sample of purified DNA was fragmented and used to construct short-insert libraries according to the manufacturer’s manual (Illumina Inc., San Diego, CA, USA). The plastome was sequenced by using the Illumina HiSeq 2000 platform at BGI. For each species, approximately four million 90 bp paired-end reads from a 500 bp insert library were produced.

### Reads filtering, plastome assembly and annotation

The BGI sequencing platform was used for preliminary filtering of raw reads. We used the NGS QC Toolkit^70^ for further quality control, and 2.39% to 7.73% of low-quality reads were rejected. Then, high-quality paired-end reads were *de novo* assembled by using the CLC Genomic Workbench v7.0.3 (CLC Inc., Arhus, Denmark) with the default parameters (word size = 64). A single contig with the full-length consensus sequence was acquired for each of the 10 sampled species. The accuracy of the assembled plastomes was further verified by using SOAPdenovo2^71^. The contigs were then aligned with the reference plastome of *Cryptomeria japonica* (GenBank accession no. NC_010548) by using local BLAST, and aligned contigs were ordered by using Geneious R8 (http://www.geneious.com/)^72^ according to overlaps and the reference plastome. To further validate the plastome assembly, Illumina paired-end reads were mapped onto the consensus sequences by using Bowtie v2.0.0-beta4^73^. Discrepancies were corrected according to the majority of mapped reads.

The plastomes were initially annotated by using Dual Organellar GenoMe Annotator (DOGMA)^74^. The exact boundaries of the annotated genes were manually verified by alignment with orthologs in other published gymnosperm plastomes. The tRNA genes were further determined by using tRNAscan-SE (http://lowelab.ucsc.edu/tRNAscan-SE/)^75^. The 10 annotated plastome sequences have been deposited in GenBank (accession nos KX832620-KX832629).

### Plastome sequence alignment, concatenation and phylogenetic analysis

The 82 protein-coding genes shared by 22 annotated plastomes were extracted by using bedtools^76^. The codon-based alignment of each gene involved use of MUSCLE^77^ implemented in MEGA v6.06^78^. Then, 82 aligned genes were concatenated to one supermatrix (PCG with 81,423 nt).

The PCG matrix was used for primary phylogenetic inference. Maximum likelihood (ML) analysis involved use of RAxML v8.1.11^79^ at the XSEDE Teragrid of the CIPRES science Gateway (http://www.phylo.org/)^80^, including tree robustness assessment with 1,000 rapid bootstrap replicates with the GTRGAMMA substitution model.

### Data filtering and modifying, evolutionary model selection and coalescent phylogenetic reconstruction

A series of strategies including data filtering and modifying, evolutionary model selection and coalescent phylogenetic reconstruction were used to alleviate the potential effect of the LBA artifact on phylogenetic reconstruction.

#### Data filtering and modifying

We used a series of data filtering and modifying of the PCG matrix (1) using only the first and second codon positions (the “PCG12” matrix); (2) using amino acid data (the “AA” matrix) (ProtTest v3^81^ was used to infer the optimal model of sequence evolution under Bayesian information criterion); and (3) removing unreliable aligned regions. Gblocks v0.91b^51^,^82^ was used to filter the unreliable aligned regions. Relaxed, default and strict parameters (“Minimum Number of Sequences for a Conserved Positions” = 11/11/21, “Minimum Number of Sequences for a Flank Position” = 11/17/21, “Maximum Number of Contiguous Nonconserved Positions” = 10/8/5, “Minimum Length of a Block” = 5/10/50 and “Allowed Gap Positions” = “With Half/None/None”) were used to produce the “PCG-GB-relaxed/default/strict” matrices, respectively.

Fourth, we excluded substitution saturation genes. The substitution saturation index (Iss) defined by Xia *et al*.^83^ was used to determine the potential phylogenetic signal for each of 82 aligned PCGs by using DAMBE v6.1.9^84^. If the value of Iss is lower than that of Iss.c for a symmetrical and an asymmetrical tree, the orthologous genes have not experienced substitution saturation and contain a phylogenetic signal for recovering true evolutionary relationships^83^. The “PCG-Iss-9” matrix comprised 9 saturated genes with Iss values significantly higher than that of Iss.c (Supplementary Table S2). In addition, the linear regression of Patristic distance and uncorrected *p* distance was used to determine the degree of substitution saturation for each of 82 PCGs by using TreSpEx v1.1^85^. Density distributions of the slope or R^2^ value were plotted by using R v3.2.2 (R Development Core Team 2012). Genes located in left shoulders of the slope or R^2^ density plots were considered saturated. The “PCG-slope-12” and “PCG-R^2^-11” matrices were built by using 12 and 11 detected saturated genes (Supplementary Fig. S2). The “PCG-Iss-73”, “PCG-slope-70” and “PCG-R^2^-71” matrices were built by removing saturated genes detected in these two analyses.

Fifth, we built rate-partitioned data subsets. Observed variability (OV)^86^ and Tree Independent Generation of Evolutionary Rates (TIGER)^87^ methods were used to measure the relative evolutionary rate for each of the aligned nucleotide sites in PCG. Using methods previously described^32^,^54^,^61^,^63^, we explored the distribution of signal in PCG by separating the moderately evolving sites from the slowly and rapidly evolving sites (Supplementary Fig. S3). First, we sorted all parsimony-informative sites (PISs) in a concatenated dataset based on estimated evolutionary rate. Then, we divided these PISs into three partitions with equal number of PISs (i.e. slow, medium and fast partitions). The sites in the second tertiles (moderately evolving sites) were used for phylogenetic reconstruction and the sites in the first and third tertiles (slowly and rapidly evolving sties) were used for comparison. Next, all parsimony uninformative sites (PUIS) and constant sites were included in each of three partitions for proper model estimation. The “PCG-OV-slow/medium/fast-partition” matrices and the “PCG-TIGER-slow/medium/fast-partition” matrices were finally obtained.

Sixth, we removed the most rapidly evolving sites. We used OV sorting as described^86^ to rank the PCG matrix from the mostly varied sites to the mostly conserved sites based on measuring character state variation in each alignment position. Then a series of alignments was generated by successively removing the mostly varied sites from the OV-sorted alignment in increments of 250. For each step, two data partitions were obtained: (1) “A” partitions comprising the shortened OV-sorted alignment and (2) “B” partitions containing the mostly varied sites. After model fitting for each partition by using ModelTest^88^, the ML distances for A and B partitions and the uncorrected *p* distances for B partitions were calculated by using PAUP*^89^. Two Pearson correlation analyses of pairwise distances were conducted at each step, correlating the 1) ML distances for A and B partitions and 2) ML and uncorrected *p* distances for B partitions (Supplementary Fig. S4b). The stopping point for site removal was determined as the point at which the two correlations showed a significant increase^86^. A sharp increase in Pearson correlation coefficient (*r*) occurred when removing 3,750 sites (position 77,673), and stopped when removing 4,250 sites (position 77,173) (Supplementary Fig. S4b). Goremykin *et al*.^86^ suggested that varied sites be removed until the end of the sharp increase in these two correlation analyses. Therefore, the optimal stopping point for varied site removal was identified at position 77,173 (“PCG-OV-sorted”) (Supplementary Fig. S4b). The ML phylogenetic reconstruction under a GTRGAMMA model and the bootstrap support measurement of alternative hypotheses, HCX (J, C), J (C, HCX) and C (J, HCX), were performed for each partition (Supplementary Fig. S4a). Supplementary Fig.S4a shows that HCX (J, C) was highly supported by including 1,750 mostly varied sites (position 79,673). J (C, HCX) was strongly supported by removing 2,250 (position 71,973) to 6,000 (position 75,423) mostly varied sites (position 71,973).

#### Evolutionary model selection

PhyloBayes v3.2^90^ was used to build Bayesian inference trees (PCG-GTR-CAT) for PCG with a site-heterogeneous mixture model, GTR-CAT^67^. Two independent Markov chain Monte Carlo chains were run for 45,600 cycles. The first 20% cycles were discarded as burn-in. Convergence of these two runs was determined when the discrepancies reached 0.3 (maxdiff < 0.3) and the minimum effective size was larger than 50.

#### Coalescent phylogenetic reconstruction

For the coalescent method, an individual gene tree for each of 82 PCGs was inferred by using RAxML with the GTRGAMMA substitution model. Species trees were reconstructed with the 82 estimated gene trees by using ASTRAL (PCG-ASTRAL)^91^,^92^.

### Divergence time estimations

Dating analyses involved a relaxed clock method, penalized likelihood^93^ in treePL^94^, which could smooth rate changes between adjacent branches of the phylogeny by applying a penalty^93^. The ML trees with branch length generated by RAxML analysis of the PCG-Iss-73 and PCG matrices were used as the input trees. This method applied a smoothing parameter, λ, to determine the magnitude of the penalty. The optimal smoothing value was determined empirically by using cross-validation^93^,^94^. For the trees from the PCG-Iss-73 and PCG matrices, the cross-validation tested 13 smoothing values separated by one order of magnitude, starting at 1 × 10^−6^. Furthermore, the minimum age of the Cupressaceae crown node was constrained at 157.2 Ma as described^35^. The maximum age of Cupressaceae crown node was assigned as 218 Ma, the estimated mean age of the stem lineage of Cupressaceae^35^. In addition, minimum age constraints from fossil data^35^ were applied to another four nodes within Cupressaceae. The detailed fossil calibrations for the five nodes are in Supplementary Table S3. We generated 1,000 ML bootstrap trees with branch lengths by using RAxML. The minimum and maximum age for the internal nodes were calculated from dating 1,000 bootstrap trees by using treePL and TreeAnnotator v1.8.4^95^.

## Additional Information

**Accession codes:** KX832620-KX832629

**How to cite this article:** Qu, X.-J. *et al*. Multiple measures could alleviate long-branch attraction in phylogenomic reconstruction of Cupressoideae (Cupressaceae). *Sci. Rep.* **7**, 41005; doi: 10.1038/srep41005 (2017).

**Publisher's note:** Springer Nature remains neutral with regard to jurisdictional claims in published maps and institutional affiliations.

## Supplementary Material

Supplementary Information

## Figures and Tables

**Table 1:** Sequence characteristics of the applied data matrices.

Datasets	No. of sites (bp)	No. of variable sites (bp)	No. of parsimonious-informative sites (bp)
PCG	81,423	17,882	11,394
PCG12	54,282	9,596	6,055
AA	27,141	7,613	5,117
PCG-GB-relaxed	74,528	16,278	10,765
PCG-GB-default	68,588	12,797	8,345
PCG-GB-strict	63,299	9,900	6,227
PCG-Iss-9	28,494	10,867	7,160
PCG-Iss-73	52,929	7,015	4,234
PCG-slope-12	28,995	11,045	7,290
PCG-slope-70	52,428	6,837	4,104
PCG-R^2^-11	19,362	7,801	5,215
PCG-R^2^-71	62,061	10,081	6,179
PCG-OV-slow-partition	10,286	10,286	3,798
PCG-OV-medium-partition	10,286	10,286	3,798
PCG-OV-fast-partition	10,286	10,286	3,798
PCG-TIGER-slow-partition	10,286	10,286	3,798
PCG-TIGER-medium-partition	10,286	10,286	3,798
PCG-TIGER-fast-partition	10,286	10,286	3,798
PCG-OV-sorted	77,173	13,632	7,810

PCG, PCG12, AA, PCG-GB-relaxed, PCG-GB-default, PCG-GB-strict, PCG-Iss-9, PCG-Iss-73, PCG-slope-12, PCG-slope-70, PCG-R^2^-11, PCG-R^2^-71, PCG-OV-slow-partition, PCG-OV-medium-partition, PCG-OV-fast-partition, PCG-TIGER-slow-partition, PCG-TIGER-medium-partition, PCG-TIGER-fast-partition and PCG-OV-sorted are explained in Materials and Methods.

**Table 2:** Estimated divergence time for the Cupressoideae clades based on the PCG-Iss-73 matrix.

Clade	PL point (Ma)	PL min (Ma)	PL max (Ma)
Crown of Cupressoideae	101.53	97.08	105.87
Crown of *Thuja-Thujopsis* clade	58.50	58.50	58.50
MRCA of *Chamaecyparis* and *Juniperus*	98.60	94.42	103.54
Crown of *Chamaecyparis-Fokienia* clade	61.10	61.10	61.10
MRCA of *Platycladus* and *Juniperus*	76.39	71.67	80.61
MRCA of *Platycladus* and *Calocedrus*	59.78	54.27	65.34
Crown of J-C-HCX clade	50.31	46.84	53.34
Crown of *Juniperus*	33.80	33.80	33.80
Crown of C-HCX subclade	46.16	42.45	50.24
Crown of HCX subclade	18.21	14.88	22.01
MRCA of *Hesperocyparis* and *Callitropsis*	15.76	11.68	18.87

Penalized likelihood (PL) point represents the point age estimation in the maximum likelihood best tree. PL min and PL max represent the minimum and maximum estimations by 1,000 maximum likelihood bootstrap trees. Ma: million years ago. MRCA: most recent common ancestor. J-C-HCX: *Juniperus-Cupressus-Hesperocyparis-Callitropsis-Xanthocyparis*.
