# Supplementary material for: Multiple measures could alleviate long-branch attraction in phylogenomic reconstruction of Cupressoideae (Cupressaceae)
Source: Sci Rep. 2017 Jan 25;7:41005. doi: 10.1038/srep41005 (PMC5264392; doi:10.1038/srep41005)
Supplement: Supplementary Information [file srep41005-s1.pdf]

Supplementary Figures and Tables

Multiple measures could alleviate long-branch attraction in phylogenomic reconstruction of Cupressoideae (Cupressaceae)

Xiaojian Qu[1,2], Jianjun Jin[1,2], Dezhu Li[1*] & Tingshuang Yi[1*]

[1]Plant Germplasm and Genomics Center, Germplasm Bank of Wild Species, Kunming Institute of Botany, Chinese Academy of Sciences, Kunming, Yunnan, 650201, China, [2]Kunming College of Life Sciences, University of Chinese Academy of Sciences, Kunming, Yunnan, 650201, China.

[*]Correspondence and requests for materials should be addressed to D.L. (email: dzl@mail.kib.ac.cn) and T.Y. (email: tingshuangyi@mail.kib.ac.cn).

Supplementary Figure S1. Phylogenies of Cupressoideae inferred from multiple maximum likelihood (ML) analyses under GTRGAMMA model. Support for the branches was estimated from 1,000 bootstrapping replicates. The bootstrap values are shown on branches, and bootstrap values of 100% are not shown. The trees are from analyses of PCG12, AA, PCG-GB-relaxed, PCG-GB-default, PCG-GB-strict, PCG-Iss-9, PCG-slope-12, PCG-slope-70, PCG-R^2-11, PCG-R^2-71, PCG-OV-slow-partition, PCG-OV-fast-partition, PCG-OV-medium-partition, PCG-TIGER-slow-partition, PCG-TIGER-fast-partition, PCG-TIGER-medium-partition, PCG-OV-sorted, PCG-GTR-CAT and PCG-ASTRAL.

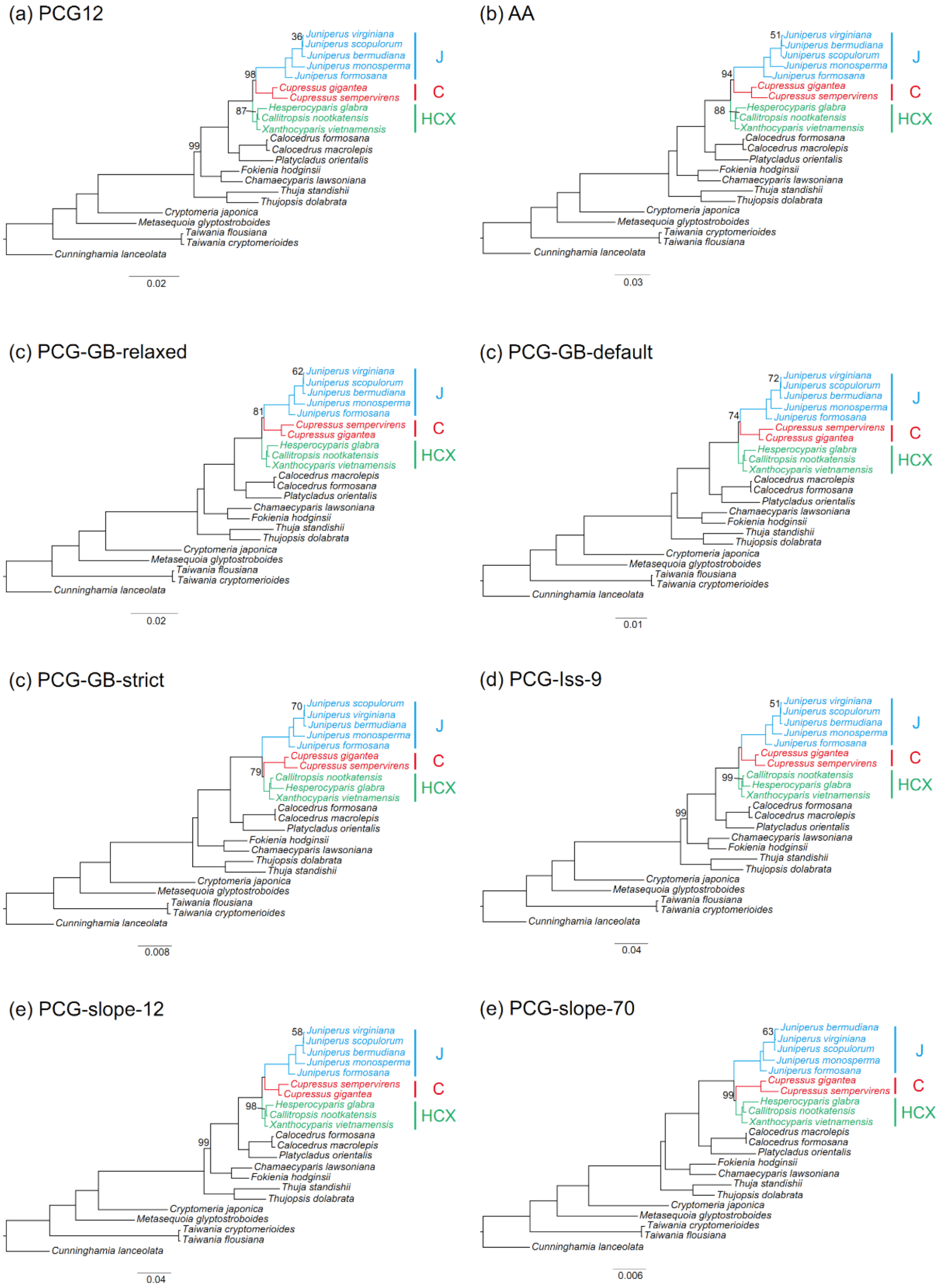
(a) PCG12
36 Juniperus virginiana
Juniperus scopulorum
Juniperus bermudiana
Juniperus monosperma
Juniperus formosana
98
Cupressus gigantea
Cupressus sempervirens
87 Hesperocyparis glabra
Callitropsis nootkatensis
Xanthocyparis vietnamensis
99
Calocedrus formosana
Calocedrus macrolepis
Platycladus orientalis
Fokienia hodginsii
Chamaecyparis lawsoniana
Thuja standishii
Thujopsis dolabrata
Cryptomeria japonica
Metasequoia glyptostroboides
Taiwania flousiana
Taiwania cryptomerioides
Cunninghamia lanceolata
0.02
J
C
HCX
(b) AA
51 Juniperus virginiana
Juniperus bermudiana
Juniperus scopulorum
Juniperus monosperma
Juniperus formosana
94
Cupressus gigantea
Cupressus sempervirens
88 Hesperocyparis glabra
Callitropsis nootkatensis
Xanthocyparis vietnamensis
Calocedrus formosana
Calocedrus macrolepis
Platycladus orientalis
Fokienia hodginsii
Chamaecyparis lawsoniana
Thuja standishii
Thujopsis dolabrata
Cryptomeria japonica
Metasequoia glyptostroboides
Taiwania cryptomerioides
Taiwania flousiana
Cunninghamia lanceolata
0.03
J
C
HCX
(c) PCG-GB-relaxed
62 Juniperus virginiana
Juniperus scopulorum
Juniperus bermudiana
Juniperus monosperma
Juniperus formosana
81
Cupressus sempervirens
Cupressus gigantea
Hesperocyparis glabra
Callitropsis nootkatensis
Xanthocyparis vietnamensis
Calocedrus macrolepis
Calocedrus formosana
Platycladus orientalis
Chamaecyparis lawsoniana
Fokienia hodginsii
Thuja standishii
Thujopsis dolabrata
Cryptomeria japonica
Metasequoia glyptostroboides
Taiwania flousiana
Taiwania cryptomerioides
Cunninghamia lanceolata
0.02
J
C
HCX
(c) PCG-GB-default
72 Juniperus virginiana
Juniperus scopulorum
Juniperus bermudiana
Juniperus monosperma
Juniperus formosana
74
Cupressus sempervirens
Cupressus gigantea
Hesperocyparis glabra
Callitropsis nootkatensis
Xanthocyparis vietnamensis
Calocedrus macrolepis
Calocedrus formosana
Platycladus orientalis
Chamaecyparis lawsoniana
Fokienia hodginsii
Thuja standishii
Thujopsis dolabrata
Cryptomeria japonica
Metasequoia glyptostroboides
Taiwania flousiana
Taiwania cryptomerioides
Cunninghamia lanceolata
0.01
J
C
HCX
(c) PCG-GB-strict
70 Juniperus scopulorum
Juniperus virginiana
Juniperus bermudiana
Juniperus monosperma
Juniperus formosana
Cupressus gigantea
Cupressus sempervirens
79
Callitropsis nootkatensis
Hesperocyparis glabra
Xanthocyparis vietnamensis
Calocedrus formosana
Calocedrus macrolepis
Platycladus orientalis
Fokienia hodginsii
Chamaecyparis lawsoniana
Thujopsis dolabrata
Thuja standishii
Cryptomeria japonica
Metasequoia glyptostroboides
Taiwania flousiana
Taiwania cryptomerioides
Cunninghamia lanceolata
0.008
J
C
HCX
(d) PCG-Iss-9
51 Juniperus virginiana
Juniperus scopulorum
Juniperus bermudiana
Juniperus monosperma
Juniperus formosana
Cupressus gigantea
Cupressus sempervirens
99
Callitropsis nootkatensis
Hesperocyparis glabra
Xanthocyparis vietnamensis
99
Calocedrus formosana
Calocedrus macrolepis
Platycladus orientalis
Chamaecyparis lawsoniana
Fokienia hodginsii
Thuja standishii
Thujopsis dolabrata
Cryptomeria japonica
Metasequoia glyptostroboides
Taiwania flousiana
Taiwania cryptomerioides
Cunninghamia lanceolata
0.04
J
C
HCX
(e) PCG-slope-12
58 Juniperus virginiana
Juniperus scopulorum
Juniperus bermudiana
Juniperus monosperma
Juniperus formosana
Cupressus sempervirens
Cupressus gigantea
98
Hesperocyparis glabra
Callitropsis nootkatensis
Xanthocyparis vietnamensis
99
Calocedrus formosana
Calocedrus macrolepis
Platycladus orientalis
Chamaecyparis lawsoniana
Fokienia hodginsii
Thuja standishii
Thujopsis dolabrata
Cryptomeria japonica
Metasequoia glyptostroboides
Taiwania cryptomerioides
Taiwania flousiana
Cunninghamia lanceolata
0.04
J
C
HCX
(e) PCG-slope-70
63 Juniperus bermudiana
Juniperus virginiana
Juniperus scopulorum
Juniperus monosperma
Juniperus formosana
Cupressus gigantea
Cupressus sempervirens
99
Hesperocyparis glabra
Callitropsis nootkatensis
Xanthocyparis vietnamensis
Calocedrus macrolepis
Calocedrus formosana
Platycladus orientalis
Fokienia hodginsii
Chamaecyparis lawsoniana
Thuja standishii
Thujopsis dolabrata
Cryptomeria japonica
Metasequoia glyptostroboides
Taiwania cryptomerioides
Taiwania flousiana
Cunninghamia lanceolata
0.006
J
C
HCX

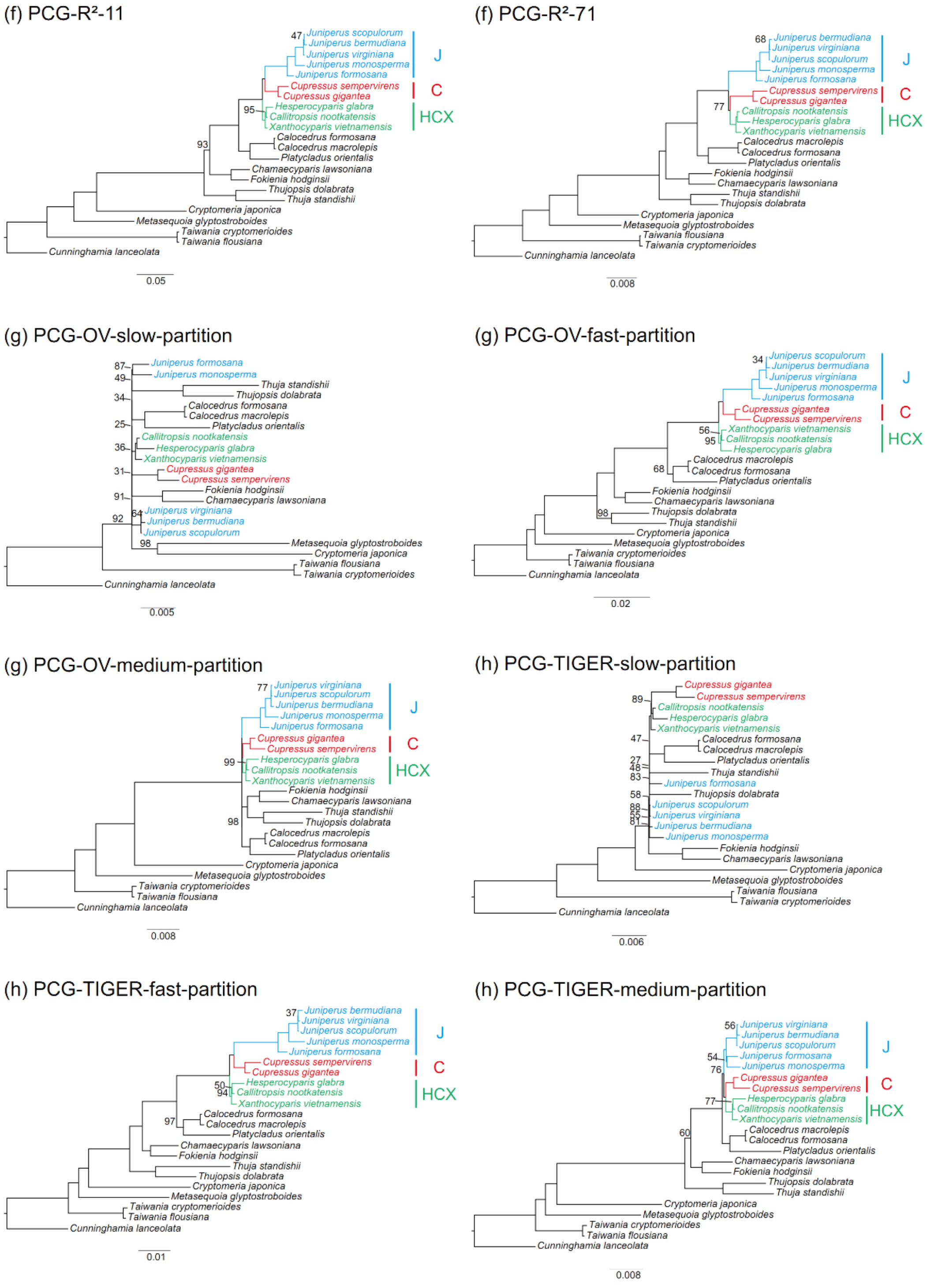
(f) PCG-R²-11
47
Juniperus scopulorum
Juniperus bermudiana
Juniperus virginiana
Juniperus monosperma
Juniperus formosana
J
Cupressus sempervirens
Cupressus gigantea
C
95
Hesperocyparis glabra
Callitropsis nootkatensis
Xanthocyparis vietnamensis
HCX
93
Calocedrus formosana
Calocedrus macrolepis
Platycladus orientalis
Chamaecyparis lawsoniana
Fokienia hodginsii
Thujopsis dolabrata
Thuja standishii
Cryptomeria japonica
Metasequoia glyptostroboides
Taiwania cryptomerioides
Taiwania flousiana
Cunninghamia lanceolata
0.05
(f) PCG-R²-71
68
Juniperus bermudiana
Juniperus virginiana
Juniperus scopulorum
Juniperus monosperma
Juniperus formosana
J
Cupressus sempervirens
Cupressus gigantea
C
77
Callitropsis nootkatensis
Hesperocyparis glabra
Xanthocyparis vietnamensis
HCX
Calocedrus macrolepis
Calocedrus formosana
Platycladus orientalis
Fokienia hodginsii
Chamaecyparis lawsoniana
Thuja standishii
Thujopsis dolabrata
Cryptomeria japonica
Metasequoia glyptostroboides
Taiwania flousiana
Taiwania cryptomerioides
Cunninghamia lanceolata
0.008
(g) PCG-OV-slow-partition
87
49
Juniperus formosana
Juniperus monosperma
34
Thuja standishii
Thujopsis dolabrata
25
Calocedrus formosana
Calocedrus macrolepis
Platycladus orientalis
36
Callitropsis nootkatensis
Hesperocyparis glabra
Xanthocyparis vietnamensis
31
Cupressus gigantea
Cupressus sempervirens
91
Fokienia hodginsii
Chamaecyparis lawsoniana
92
64
Juniperus virginiana
Juniperus bermudiana
Juniperus scopulorum
98
Metasequoia glyptostroboides
Cryptomeria japonica
Taiwania flousiana
Taiwania cryptomerioides
Cunninghamia lanceolata
0.005
(g) PCG-OV-fast-partition
34
Juniperus scopulorum
Juniperus bermudiana
Juniperus virginiana
Juniperus monosperma
Juniperus formosana
J
Cupressus gigantea
Cupressus sempervirens
C
56
95
Xanthocyparis vietnamensis
Callitropsis nootkatensis
Hesperocyparis glabra
HCX
68
Calocedrus macrolepis
Calocedrus formosana
Platycladus orientalis
Fokienia hodginsii
Chamaecyparis lawsoniana
98
Thujopsis dolabrata
Thuja standishii
Cryptomeria japonica
Metasequoia glyptostroboides
Taiwania cryptomerioides
Taiwania flousiana
Cunninghamia lanceolata
0.02
(g) PCG-OV-medium-partition
77
Juniperus virginiana
Juniperus scopulorum
Juniperus bermudiana
Juniperus monosperma
Juniperus formosana
J
Cupressus gigantea
Cupressus sempervirens
C
99
Hesperocyparis glabra
Callitropsis nootkatensis
Xanthocyparis vietnamensis
HCX
Fokienia hodginsii
Chamaecyparis lawsoniana
98
Thuja standishii
Thujopsis dolabrata
Calocedrus macrolepis
Calocedrus formosana
Platycladus orientalis
Cryptomeria japonica
Metasequoia glyptostroboides
Taiwania cryptomerioides
Taiwania flousiana
Cunninghamia lanceolata
0.008
(h) PCG-TIGER-slow-partition
Cupressus gigantea
Cupressus sempervirens
89
Callitropsis nootkatensis
Hesperocyparis glabra
Xanthocyparis vietnamensis
47
Calocedrus formosana
Calocedrus macrolepis
27
48
83
Platycladus orientalis
Thuja standishii
Juniperus formosana
58
Thujopsis dolabrata
88
55
81
Juniperus scopulorum
Juniperus virginiana
Juniperus bermudiana
Juniperus monosperma
Fokienia hodginsii
Chamaecyparis lawsoniana
Cryptomeria japonica
Metasequoia glyptostroboides
Taiwania flousiana
Taiwania cryptomerioides
Cunninghamia lanceolata
0.006
(h) PCG-TIGER-fast-partition
37
Juniperus bermudiana
Juniperus virginiana
Juniperus scopulorum
Juniperus monosperma
Juniperus formosana
J
Cupressus sempervirens
Cupressus gigantea
C
50
94
Hesperocyparis glabra
Callitropsis nootkatensis
Xanthocyparis vietnamensis
HCX
97
Calocedrus formosana
Calocedrus macrolepis
Platycladus orientalis
Chamaecyparis lawsoniana
Fokienia hodginsii
Thuja standishii
Thujopsis dolabrata
Cryptomeria japonica
Metasequoia glyptostroboides
Taiwania cryptomerioides
Taiwania flousiana
Cunninghamia lanceolata
0.01
(h) PCG-TIGER-medium-partition
56
Juniperus virginiana
Juniperus bermudiana
Juniperus scopulorum
54
Juniperus formosana
Juniperus monosperma
J
76
Cupressus gigantea
Cupressus sempervirens
C
77
Hesperocyparis glabra
Callitropsis nootkatensis
Xanthocyparis vietnamensis
HCX
60
Calocedrus macrolepis
Calocedrus formosana
Platycladus orientalis
Chamaecyparis lawsoniana
Fokienia hodginsii
Thujopsis dolabrata
Thuja standishii
Cryptomeria japonica
Metasequoia glyptostroboides
Taiwania cryptomerioides
Taiwania flousiana
Cunninghamia lanceolata
0.008

(i) PCG-OV-sorted

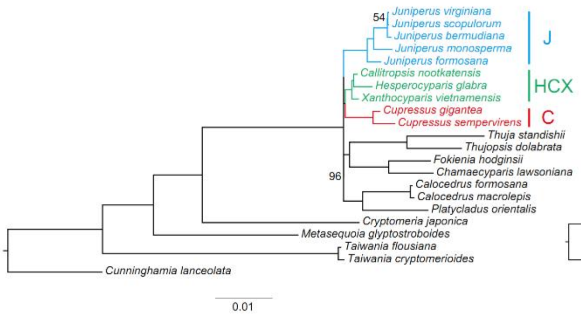

(j) PCG-GTR-CAT

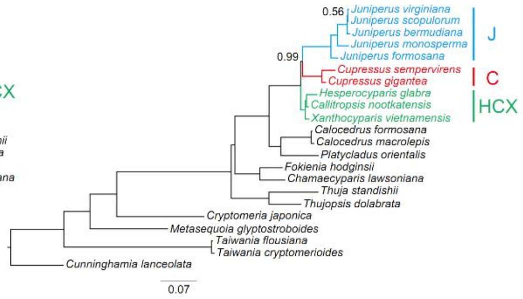

(k) PCG-ASTRAL

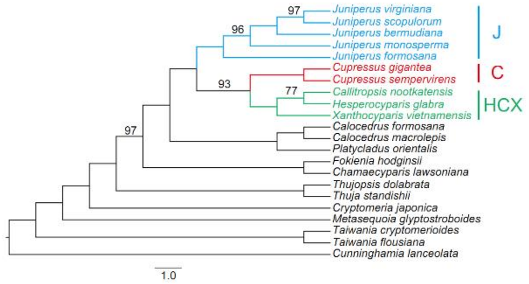

Supplementary Figure S2. Density plots of gene-specific saturation indices for the 82 PCG genes. (a) Slopes of the linear regression between patristic and uncorrected pairwise distances. (b) R^2 for the linear regression between patristic and uncorrected pairwise distances. Dashed line indicates the starting shoulder value. Red areas in the left-hand side are genes that might be saturated.

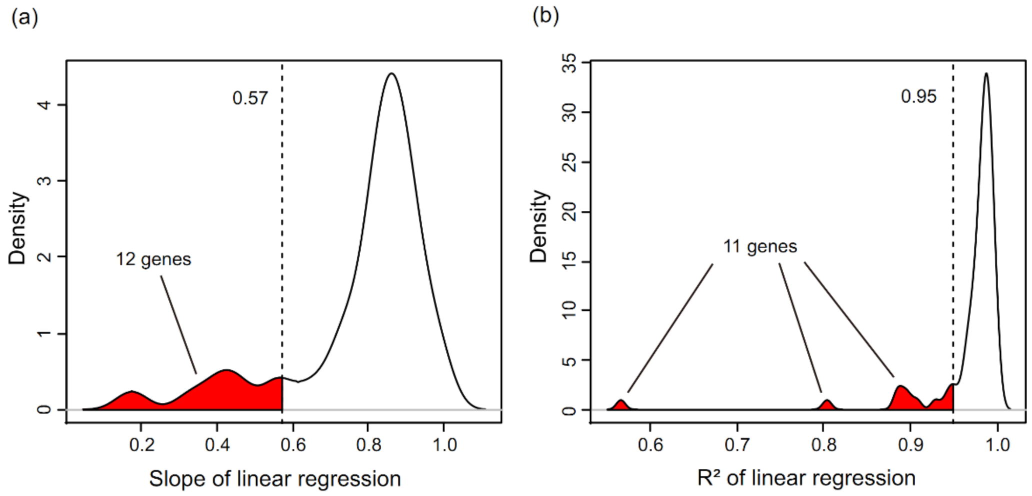

Supplementary Figure S3. Estimated evolutionary rates for nucleotide sites in the concatenated PCG data set. Parsimony informative sites in the concatenated dataset were sorted by the Observed Variability (OV) method or the Tree Independent Generation of Evolutionary Rates (TIGER) method and divided into three equal partitions.

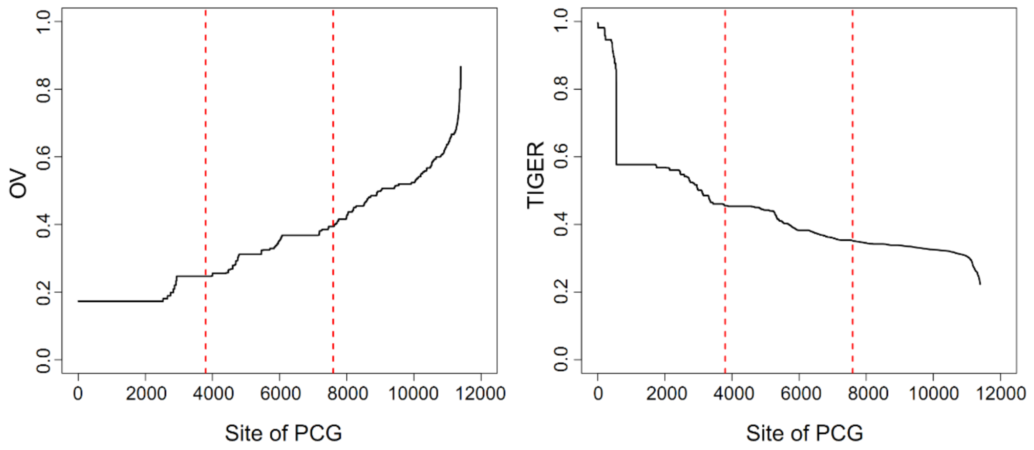

Supplementary Figure S4. OV-sorting analysis. (a) Bootstrap support in ML phylogenetic reconstruction for three alternative relationships. Digits on the y-axis indicate bootstrap support values, and on the x-axis indicate length of A partitions. (b) Pearson correlation analyses. The blue dotted line indicates the Pearson correlation coefficient (*r*) of ML distances for A (conserved) and B (varied) partitions. The red dotted line represents the *r* value for uncorrected *p* distances and ML distances for the B partition. The optimal stopping point for varied site removal was identified at position 77,173. Digits on the y-axis indicate Pearson correlation coefficient and digits on the x-axis indicate length of A partitions.

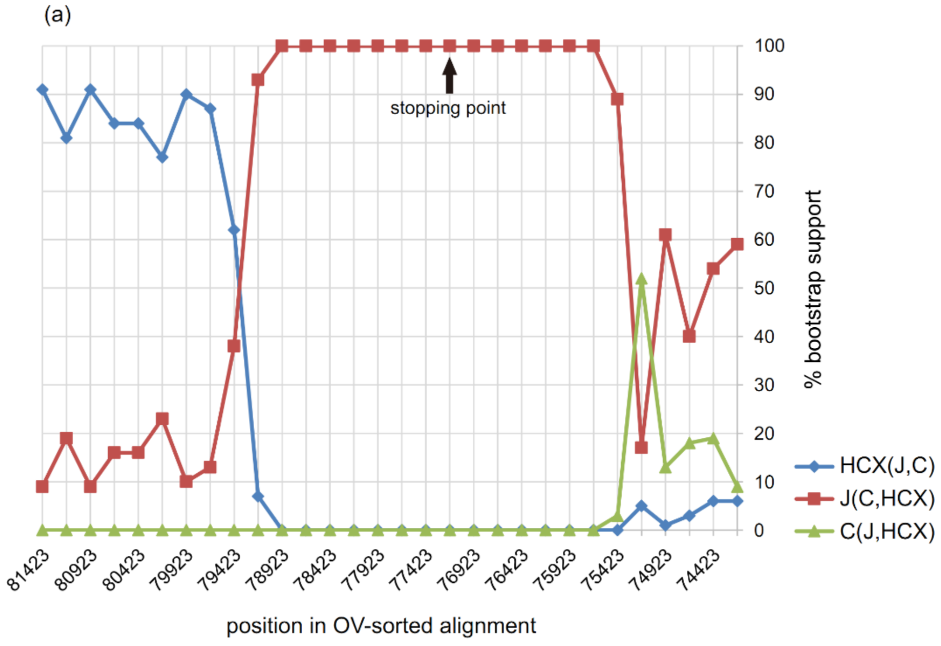

(a)
stopping point
% bootstrap support
100
90
80
70
60
50
40
30
20
10
0
HCX(J,C)
J(C,HCX)
C(J,HCX)
81423
80923
80423
79923
79423
78923
78423
77923
77423
76923
76423
75923
75423
74923
74423
position in OV-sorted alignment

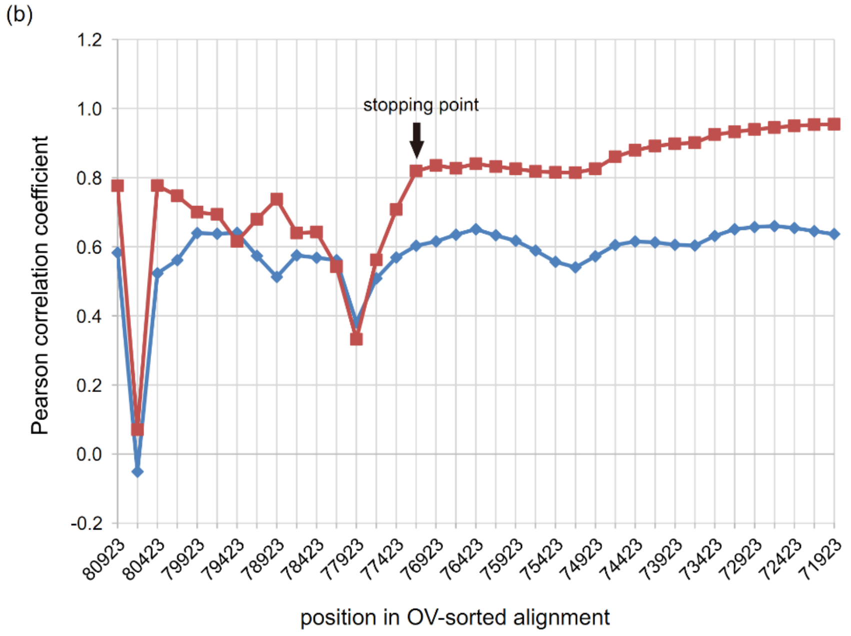

(b)
stopping point
Pearson correlation coefficient
1.2
1.0
0.8
0.6
0.4
0.2
0.0
-0.2
80923
80423
79923
79423
78923
78423
77923
77423
76923
76423
75923
75423
74923
74423
73923
73423
72923
72423
71923
position in OV-sorted alignment

Supplementary Figure S5. Chronogram of Cupressoideae based on the PCG matrix inferred by using treePL. Blue bars represent the minimum and maximum estimation of node ages. Red dots represent fossil calibration points in Supplementary Table S3. Red line indicates the Cretaceous/Tertiary boundary.

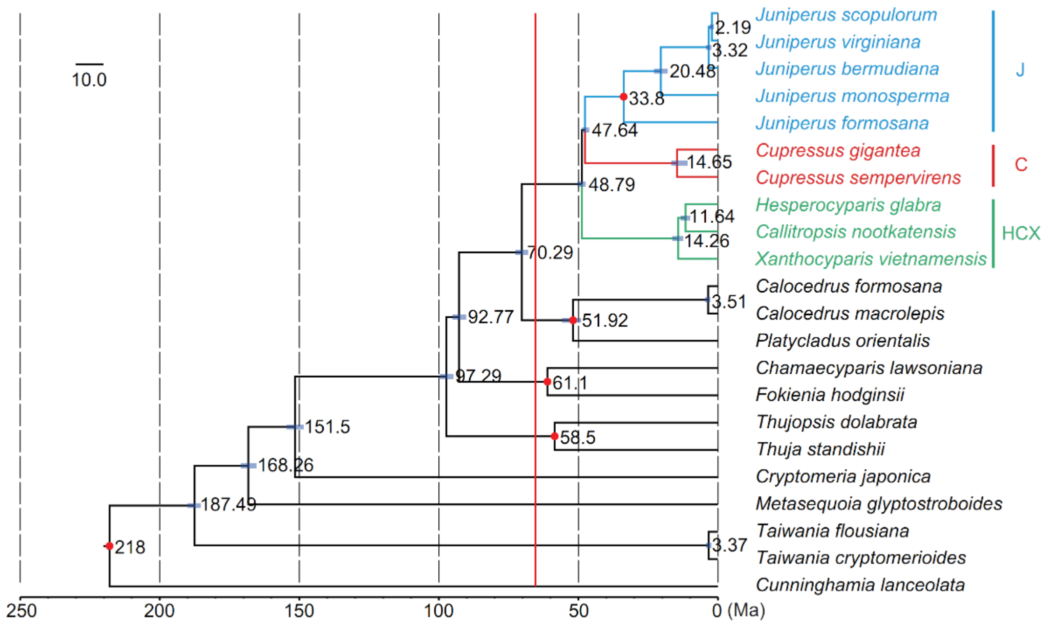

Supplementary Table S1. Cupressaceae taxa sampled in this study. The plastome sequences from KX832620 to KX832629 were generated in this study.

Subfamily	Species	Accession no.	Collection no.
Cupressoideae	*Callitropsis nootkatensis*	NC_026295	-
	Calocedrus formosana	KX832620	Yi16045
	Calocedrus macrolepis	KX832621	Yi16046
	Chamaecyparis lawsoniana	KX832622	Yi16050
	Cupressus gigantea	NC_028155	-
	Cupressus sempervirens	NC_026296	-
	Fokienia hodginsii	KX832623	Yi16052
	Hesperocyparis glabra	KX832624	Yi16053
	Juniperus bermudiana	NC_024021	-
	Juniperus formosana	KX832625	Yi16054
	Juniperus monosperma	NC_024022	-
	Juniperus scopulorum	NC_024023	-
	Juniperus virginiana	NC_024024	-
	Platycladus orientalis	KX832626	Yi16058
	Thuja standishii	KX832627	Yi16059
	Thujopsis dolabrata	KX832628	Yi16060
	Xanthocyparis vietnamensis	KX832629	Yi16061
Cunninghamioideae	*Cunninghamia lanceolata*	NC_021437	-
Sequoioideae	*Metasequoia glyptostroboides*	NC_027423	-
Taiwanioideae	*Taiwania cryptomerioides*	NC_016065	-
	Taiwania flousiana	NC_021441	-
Taxodioideae	*Cryptomeria japonica*	NC_010548	-

Supplementary Table S2. Index of substitutuion saturation (Iss) values of 82 protein-coding genes for 22 Cupressaceae species. Iss: an entropy-based index of substitution saturation. Iss.c: the critical value of Iss. *: Iss value was greater than Iss.c value.

Order	Gene	Iss	Iss.c (symmetrical tree)	*P*-value	Iss.c (asymmetrical tree)	*P*-value
1	*petN*	0.0142	0.4176	0	0.3619	0
2	*psbL*	0.0156	0.4865	0	0.3555	0
3	*petG*	0.0167	0.4802	0	0.3555	0
4	*psbN*	0.0169	0.5144	0	0.3571	0
5	*rbcL*	0.0366	0.7827	0	0.563	0
6	*psbI*	0.0379	0.4736	0	0.3556	0
7	*ycf3*	0.0382	0.7151	0	0.4645	0
8	*psaB*	0.0434	0.797	0	0.5961	0
9	*ndhE*	0.0514	0.6571	0	0.4127	0
10	*ndhA*	0.0571	0.7719	0	0.54	0
11	*ndhJ*	0.0574	0.7179	0	0.4665	0
12	*ndhB*	0.0649	0.7841	0	0.5659	0
13	*atpH*	0.0659	0.6281	0	0.3939	0
14	*psaC*	0.066	0.6281	0	0.3939	0
15	*psaA*	0.0699	0.7979	0	0.5974	0
16	*psbA*	0.0707	0.77	0	0.536	0
17	*rps7*	0.0719	0.7064	0	0.456	0
18	*psbK*	0.0748	0.5826	0	0.3727	0
19	*ndhC*	0.0777	0.6793	0	0.4302	0
20	*petA*	0.0782	0.7653	0	0.5268	0
21	*rpl14*	0.0783	0.6812	0	0.4318	0
22	*psbD*	0.0806	0.77	0	0.536	0
23	*psbB*	0.0827	0.7849	0	0.5687	0
24	*petB*	0.085	0.7395	0	0.4866	0
25	*rps8*	0.0873	0.6899	0	0.4397	0
26	*ndhH*	0.0895	0.7748	0	0.5462	0
27	*psbH*	0.0957	0.6166	0	0.3876	0
28	*atpI*	0.0966	0.7502	0	0.5009	0
29	*chlB*	0.0973	0.785	0	0.5696	0
30	*psbC*	0.0975	0.7825	0	0.5626	0
31	*rps14*	0.0985	0.6571	0	0.4127	0
32	*ycf4*	0.1007	0.7248	0	0.4719	0
33	*psbZ*	0.1026	0.5854	0	0.3738	0
34	*psbE*	0.1118	0.6351	0	0.398	0
35	*rpl36*	0.1151	0.4802	0	0.3555	0
36	*chlN*	0.116	0.7815	0	0.5604	0
37	*rpl2*	0.1253	0.7572	0	0.5121	0
38	*atpF*	0.1254	0.7254	0	0.4724	0
39	*psbF*	0.1254	0.4925	0.0001	0.3557	0.0069

40	*chlL*	0.1287	0.7601	0	0.517	0
41	*atpE*	0.1394	0.6939	0	0.4435	0
42	*rpl33*	0.1429	0.5961	0	0.378	0
43	*rps11*	0.1455	0.6899	0	0.4397	0
44	*rps15*	0.1465	0.64	0	0.401	0.0001
45	*cemA*	0.1486	0.7538	0	0.5064	0
46	*psbM*	0.1511	0.4736	0	0.3556	0
47	*atpA*	0.1533	0.7849	0	0.5686	0
48	*ndhF*	0.1613	0.7977	0	0.597	0
49	*ndhG*	0.1637	0.723	0	0.4705	0
50	*ndhI*	0.1747	0.7112	0	0.4611	0
51	*psbT*	0.1904	0.4667	0.0035	0.3559	0.0603
52	*rps4*	0.1993	0.7387	0	0.4857	0
53	*petL*	0.2013	0.4357	0.0054	0.3589	0.0495
54	*psaM*	0.2025	0.452	0	0.357	0.0079
55	*atpB*	0.2092	0.785	0	0.5691	0
56	*psaI*	0.22	0.4736	0	0.3556	0.0194
57	*matK*	0.2219	0.7852	0	0.5707	0
58	*ndhK*	0.2366	0.7552	0	0.5087	0
59	*rps2*	0.2451	0.7471	0	0.4963	0
60	*ndhD*	0.2729	0.7849	0	0.5689	0
61	*rps19*	0.2812	0.6597	0	0.4145	0.1181
62	*rpl20*	0.2895	0.6774	0	0.4286	0.0658
63	*psaJ*	0.3104	0.5193	0.1521	0.3577	0.7439
64	*rps12*	0.3325	0.6848	0.0014	0.4351	0.3414
65	*rpl23*	0.336	0.6584	0.002	0.4136	0.4495
66	*psbJ*	0.3522	0.4983	0.127	0.3559	0.969
67	*ccsA*	0.4041	0.7656	0	0.5274	0.0494
68	*infA*	0.4557	0.6446	0.0527	0.404	0.5939
69	*petD*	0.4929	0.7158	0.1791	0.465	0.8661
70	*rpoB*	0.5052	0.8089	0	0.6209	0.0042
71	*rpoA*	0.5057	0.7709	0	0.5379	0.6189
72	*rpl16*	0.6311	0.7025	0.6574	0.452	0.2674
73	*rpoC1*	0.6989	0.7999	0.0505	0.6005	0.0568
74	*rpoC2**	0.7345	0.8095	0.0308	0.6262	0.0019
75	*ycf2**	0.8376	0.8389	0.9496	0.6405	0
76	*rps3**	1.0073	0.7518	0.0302	0.5033	0
77	*rpl22**	1.1283	0.7172	0	0.466	0
78	*clpP**	1.1514	0.7729	0	0.542	0
79	*ycf1**	1.1606	0.8414	0	0.6404	0
80	*rpl32**	1.3362	0.6976	0	0.4471	0
81	*accD**	1.4705	0.8085	0	0.6188	0
82	*rps18**	1.9964	0.7371	0	0.4839	0

Supplementary Table S3. Fossil calibrations implemented in this study. Detailed fossil calibration for these five nodes are in Materials and Methods. Ma: million years ago. MRCA: most recent common ancestor.

**Calibration point**	**Assigned minimum age (Ma)**	**Note**
Crown of Cupressoideae	157.2	Justification by Mao *et al.*[1]
Crown of *Thuja*-*Thujopsis* clade	58.5	Justification by Mao *et al.*[1]
Crown of *Chamaecyparis*-*Fokienia* clade	61.1	Justification by Mao *et al.*[1]
MRCA of *Platycladus* and *Calocedrus*	28.3	Justification by Mao *et al.*[1]
Crown of *Juniperus*	33.8	Justification by Mao *et al.*[1]

1. Mao, K. S. *et al.* Distribution of living Cupressaceae reflects the breakup of Pangea. *Proc. Natl. Acad. Sci. USA* **109**, 7793-7798 (2012).

Supplementary Table S4. The estimated divergence time for the Cupressoideae clades based on the PCG matrix. Penalized likelihood (PL) point represents the point age estimation in the maximum likelihood best tree. PL min and PL max represent the minimum and maximum estimations from 1,000 maximum likelihood bootstrap trees. Ma: million years ago. MRCA: most recent common ancestor. J-C-HCX: *Juniperus-Cupressus-Hesperocyparis-Callitropsis-Xanthocyparis*.

**Clade**	**PL point (Ma)**	**PL min (Ma)**	**PL max (Ma)**
Crown of Cupressoideae	97.29	94.73	99.65
Crown of *Thuja-Thujopsis* clade	58.50	58.50	58.50
MRCA of *Chamaecyparis* and *Juniperus*	92.77	90.25	95.10
Crown of *Chamaecyparis-Fokienia* clade	61.10	61.10	61.10
MRCA of *Platycladus* and *Juniperus*	70.29	68.03	72.60
MRCA of *Platycladus* and *Calocedrus*	51.92	49.02	55.75
Crown of J-C-HCX clade	48.79	47.48	50.29
Crown of J-C subclade	47.64	46.16	48.87
Crown of *Juniperus*	33.80	33.80	33.80
Crown of HCX subclade	14.26	12.36	16.23
MRCA of *Hesperocyparis* and *Callitropsis*	11.64	10.05	13.30
